# Early Use of Botulinum Toxin in Post-Stroke Spasticity Has the Potential to Prevent Post-Stroke Upper Limb Pain—A Secondary Analysis of the EUBoSS Randomised Controlled Trial

**DOI:** 10.3390/toxins18030147

**Published:** 2026-03-18

**Authors:** Cameron Lindsay, Fraser Philp, Anand D. Pandyan

**Affiliations:** 1 Faculty of Health and Social Sciences, Bournemouth University, Poole BH12 5BB, UK; 2 School of Allied Health Professions and Nursing, University of Liverpool, Liverpool L69 3GB, UK; 3 Faculty of Education, Health and Wellbeing, University of Wolverhampton, Wolverhampton WV1 1LY, UK; a.pandyan@wlv.ac.uk

**Keywords:** stroke, arm, botulinum toxin, spasticity, upper limb, pain

## Abstract

Post-stroke upper limb pain is prevalent and challenging to manage once established. Early use of botulinum toxin can reduce spasticity and contracture development and has potential to prevent or reduce pain. A secondary analysis of the EUBoSS study was undertaken to report pain prevalence in people post-stroke with severe upper limb impairment and spasticity in a hyper/acute setting, identify if botulinum toxin Type-A (BoNTA) could prevent pain developing and reduce pain if already present and evaluate differences in analgesic use between BoNTA and placebo groups. Odds ratios (OR) with 95% confidence intervals (CI) were calculated. Ninety-three participants (48F:45M) were randomised at a median of 11 days post-stroke (IQR 8–19) and included in the intention-to-treat analysis. Pain prevalence increased from 29.0% (95% CI [20.1–37.9%]) to 63.4% (95% CI [54.0–72.9%]) at six months. BoNTA treatment may prevent the development of pain at six months (OR = 0.42, 95% CI [0.18 to 1.01]) but not at three months (OR = 0.57, 95% CI [0.25 to 1.32]). The odds ratio for being on at least one analgesic at six months in the BoNTA group was 0.35 ([95% 0.14 to 0.87]). This secondary analysis suggests that early treatment of spasticity with BoNTA may potentially help prevent post-stroke upper limb pain and reduce analgesic use but appears less effective once pain is established. Further prospective studies are required to verify the hypotheses generated from this secondary analysis.

## 1. Introduction

Post-stroke pain is common, with reported prevalences of all pain types and locations varying between 10% and 70% [1,2,3,4,5,6]. Nearly half of people report newly developed pain between 5 weeks and 6 months following a stroke, with the most painful point occurring in the first 100 days [1,5]. For the upper limb, approximately 36% of people post-stroke present with increasing pain profiles, characterised by high pain scores at 3 days post-stroke, with the most significant increase in pain occurring between 10 days and 3 months and peaking at 3 months [7]. Shoulder pain, once established, can be persistent with up to 20% of people reporting ongoing shoulder pain at 4 years [8]. The shoulder is the most affected, and [1] whilst pain prevalences vary due to study samples and data collection methods, at the shoulder, the reported pain prevalence is approximately 35% in the first few weeks [7,9], increasing to approximately 42% [10] to 44% [11] at 8 to 12 weeks, and then reducing to around 15 to 30% at six months [1,10]. The combined pain prevalence in the upper limb is reportedly 56% at six months, with the shoulder, fingers and elbow accounting for 27%, 16% and 13% respectively [1].

Whilst pain is multifactorial, the possible causes post-stroke include central neuropathic pain, commonly referred to as central post-stroke pain [12], pain associated with the inflammatory response to the secondary complications (e.g., subluxation and contractures or limb deformities) [13], or pain associated with spasticity or spastic dystonia [14,15]. People with reduced range of movement, severe motor and sensory impairments and spasticity post-stroke are more likely to have high and increasing pain profiles [7].

High levels of pain are associated with lower levels of independence, decreased function, quality of life and increased mortality [5,7,16]. People with increasing pain profiles after stroke are nearly five times more likely to have worse function at 12 months irrespective of age, stroke type and severity (OR = 4.99, 95% CI [1.53 to 16.30]) [7]. Appropriate pain management is important for improving the lives of people affected by stroke and possibly preventing the development or worsening of pain [17]. Existing guidelines recommend pharmacological management of pain alongside education, use of slings, positioning and neuromuscular electrical stimulation dependent on individual presentation [18,19], although evidence for their effectiveness at preventing pain or the development of secondary complications, such as contracture, that could lead to pain are limited [20].

Botulinum toxin Type-A (BoNTA) has been shown to significantly reduce spasticity-related upper limb pain at follow-up time points [21]. However, existing studies have mainly focused on chronic stroke, with most enrolling people after 3 months or more [21]. There is therefore limited evidence to inform decision-making on the role of BoNTA for both the treatment or prevention of pain in the acute or hyper acute stages.

The Early Use of Botulinum Toxin in Post Stroke Spasticity (EUBoSS) trial demonstrated, in people who had severe upper limb impairment after a stroke, that treating spasticity with BoNTA as soon as this was present reduced both spasticity and contractures and was economically viable [22,23,24]. In this current secondary analysis, we investigated whether the early use of BoNTA on post-stroke spasticity had any effect on pain. In particular, it set out to (1) understand the prevalence of pain in people who had no function and had first signs of spasticity in an hyper acute/acute population, (2) identify if treating spasticity before pain was present could prevent pain from occurring, (3) identify if treatment with botulinum toxin was able to decrease pain if already present, and (4) identify if there was any difference in the use of analgesics between the groups receiving BoNTA or the placebo.

## 2. Results

Ninety-three participants were randomised and injected. All were appropriate for inclusion in the intention-to-treat analysis, of whom 48 were male and 45 were female. For the analysis of analgesic use, the actual number analgesics and the number of people in each group for each timepoint are reported.

### 2.1. Pain Prevalence in People Post-Stroke with Severe Upper Limb Impairment and Spasticity

At baseline, a median of 11 days (IQR 8 to 19) post-stroke, 27 participants (29%) were identified as having pain in the arm (95% CI [20.1% to 37.9%] (BoNTA group (*n* = 13) and placebo group (*n* = 14)) (Table 1).

Sixty-six participants did not report pain at baseline (BoNTA group (*n* = 32) and placebo group (*n* = 34)). There were no significant differences in age, NIHSS score, Barthel Index and ARAT score between those with and without pain at baseline (Table 2).

Pain prevalence increased over the study from baseline, with 60.2% (95% CI [50.6% to 69.8%]) and 63.4% (95% CI [54.0% to 72.9%]) identified as having pain at three and six months respectively. At three months, 24 participants in the BoNTA group had pain compared to 32 in the placebo group (OR = 0.57, 95% CI [0.25 to 1.32]), in favour of the BoNTA group (Table 1). At the six-month follow-up, the same number of participants (24) were identified as having pain in the treatment group, whereas the number in the placebo group had increased to 35. The odds ratio for developing pain at six months in the BoNTA group was 0.42, 95% CI [0.18 to 1.01].

At six months post-stroke, the participants who had pain had lower NIHSS and ARAT scores, with mean differences of −3.163 and 1.119 respectively (Table 3).

### 2.2. Does Treating Spasticity Before Pain Occurs Prevent the Development of Pain?

Since many participants reported no pain prior to injection, an analysis was conducted to explore if treatment with BoNTA could potentially be used prophylactically to prevent the pain developing (Table 4).

At three months, 11 participants in the BoNTA group had developed pain compared to 18 in the placebo group (OR = 0.47, 95% CI [0.17 to 1.26]). At the end of study (six months post-injection), 11 participants in the BoNTA group reported pain compared to 21 in the placebo group (OR = 0.32, 95%CI [0.12 to 0.89]).

### 2.3. Can Botulinum Toxin Be Used to Treat Pain in People with Pain and Spasticity Early After Stroke?

The numbers who presented with both pain and spasticity in the BoNTA (*n* = 13) and placebo groups (*n* = 14) were small. There was minimal change within groups at both the three- and six-month timepoints (Table 5).

### 2.4. Are There Any Differences in Analgesic Use Between the Groups?

No participants were on pain medication prior to baseline. Baseline medication was not documented because it was liable to change frequently in the early stage of the stroke. As a result, the first medication was documented at discharge when a stable prescription had been achieved, and then at three and six months (Table 6).

At discharge, 24 participants (out of 42) in the BoNTA group were on no analgesic medication compared to 18 (out of 46) in the placebo group. The odds ratio for being on an analgesic at discharge in the BoNTA group relative to the placebo group was lower (OR = 0.49 (95% CI [0.21 to 1.13]). The odds ratio for being on at least one analgesic at three months was 0.31 (95% CI [0.13 to 0.77]). By six months, 21 participants (out of 40) in the BoNTA group were on no analgesic medication compared to 12 (out of 43) in the placebo group. The odds ratio for being on at least one analgesic at six months in the BoNTA group was 0.35 (95% CI [0.14 to 0.87]).

## 3. Discussion

The aim of this secondary analysis was to evaluate an existing dataset from the EUBoSS trial to explore the prevalence of pain in people with lower levels of arm function and explore the potential effects of early use of BoNTA in the upper limb for preventing and managing pain. Pain prevalence increased from 29.0% (95% CI [20.1% to 37.9%]) to 63.4% (95% CI [54.0% to 72.9%]), where it was highest at six months. The prevalence of pain estimated is consistent with the previous literature; however, the sample was not consistent with a general stroke population [1,2,3,4,5,6]. The study sample comprised people who had severe activity limitations in the upper limb and also had spasticity. Based on the literature, it would be expected that the estimated prevalence of spasticity would be much higher for two reasons. Firstly, previous reports have suggested that people with higher levels of disability and poor potential for recovery are more likely to report pain [6,7]. Secondly, one would expect people with spasticity to have an increased likelihood of having pain [25]. Further work is needed to explore if our current assumptions that contribute to pain are valid, particularly the relationship between activity limitations, spasticity and pain.

### 3.1. Prevention of Pain

The secondary analysis suggests that treatment, when the first direct signs of spasticity were observed, reduced the likelihood of participants in the treatment group developing pain at both three and six months post-stroke. We know that all participants who received the active treatment responded with a significant reduction in spasticity and that the treatment was effective between 6 and 12 weeks [23]. It is therefore possible that the prevention of spasticity may have prevented the development of pain. When considering the potential mechanisms by which this may have happened, it is worth noting that the treatment effects on spasticity did not extend beyond the 12 weeks. It is possible that BoNTA may have played a role in preventing the development of pain, as the effects outlasted the effects of active treatment [26,27,28]. It is also possible that the treatment effect in reducing the rate of contracture development with BoNTA may have had a secondary effect that contributed to participants not developing pain [23,26,29]. The original trial was designed to position the maximum treatment effect to capitalise on the plastic changes that occur immediately post-injury [7,16]. This may potentially explain some of the results observed in this analysis.

This secondary analysis has highlighted two hypotheses that will need to be tested in future mechanistic studies across all stages of the stroke recovery pathway and subgroups. These are (1) if BoNTA can be used prophylactically to prevent pain in people after a stroke, and (2) if pain is a secondary consequence of spasticity and/or contractures.

### 3.2. Treatment of Pain

The two mechanisms by which BoNTA treats pain are either peripherally, by its effects on sensory nerves through inhibition of neurotransmitters and inflammatory mediators, or centrally through axonal transport [30]. In patients who have established pain, it is possible that neither of these mechanisms would be sufficient for targeting pain. It was not possible to identify the factors that contributed to pain at baseline, and some people may have had factors other than stroke that contributed to their pain. In this study, the number of people who had pain prior to treatment was small, and treatment appeared not to have had an effect. We acknowledge that our sample size was not adequately powered for detecting the therapeutic effects of BoNTA.

### 3.3. Analgesic Medication Use

Participants in the BoNTA group were OR = 0.35 times (95% CI [0.14 to 0.87]) less likely to be on at least one analgesic compared to the treatment group at six months. The use of multiple analgesic as an outcome measure might not be appropriate on its own. We acknowledge that analgesics may be taken for other pain-related problems, which were unrelated to the trial intervention, and the indication for use was not confirmed. Participants may have been on other medications, such as antispasmodics, which could have had a secondary pain modulation effect [31]. However, further considerations based on the trial results are that cost utility analysis suggested that there was cost benefit to using BoNTA, and one factor responsible for the reduced cost was the reduction in the use of concomitant pain medication [23,24]. Previous studies have identified that the evaluation of pain outcomes and review of medication after initiation of analgesic treatments is low [31,32]. The reduction in the total number of pain medicines may also be likely to reduce the potential risk associated with falls, and this is a common problem for people post-stroke who are discharged into the community or the care sector [32,33,34]. Further research will be required to evaluate the therapeutic effects of BoNTA on people who have established post-stroke pain.

### 3.4. Limitations

Dichotomisation of pain outcomes was selected as a pragmatic solution to ensure the inclusion of all participants with communication or cognitive impairments. The use of pain behaviour observations by clinicians has been recommended as an alternative method for pain identification and management, although its implementation in research and clinical practice still remains limited [31,35,36]. This approach, whilst practical, results in a loss of sensitivity regarding pain intensity outcomes and may introduce another source of classification bias, highlighting the challenges of collecting pain outcomes in stroke research [5]. Despite a range of pain outcomes used in stroke or those with communication difficulties [36,37], there is currently no universally agreed outcome or method for assessment in clinical or research practice [37,38]. Current challenges to pain evaluation include timely identification and measurement, appropriateness of measures for those with cognitive and communication difficulties beyond aphasia, limited data regarding reliability and responsiveness and adaptation for use across the different timepoints, types and levels of impairment following stroke [36,37]. Further development and evaluation of methods used for the identification and measurement of pain across the stroke recovery pathway are needed.

The primary trial and current study are not powered to detect differences in secondary outcomes or further subgroup analysis. Consistent with the nature of a secondary exploratory study, the results and interpretation must be considered with this understanding. The subgroup of participants presenting with pain at baseline was small in both the treatment (*n* = 13) and placebo groups (*n* = 14), and that limits conclusions regarding the efficacy of BoNTA for treating pain during the acute phase. Whilst people in the BoNTA group had a lower odds ratio for being on at least one analgesic, the inability to confirm the indications for analgesic use limits the ability to infer that the reduction is related to post-stroke upper limb pain.

## 4. Conclusions

The secondary analysis suggests that in people with severe upper limb impairment and no pain prior to treatment, the use of BoNTA to treat spasticity, as soon as signs were observed, may have reduced the probability of developing post-stroke pain. If pain was already present prior to the treatment being administered, BoNTA appeared to have limited effect. Further prospective research that is adequately powered will now be required to verify the hypotheses generated from this study.

## 5. Materials and Methods

This is a secondary exploratory analysis of the EUBoSS controlled trial dataset. EUBoSS was a phase II, double-blind, randomised, placebo-controlled trial undertaken at a single NHS Trust. The trial protocol is published and is available online [22]. The trial was powered to detect changes in the primary outcome measure of arm function assessed using the Action Research Arm Test (ARAT) [22,23]. All patients with a diagnosis of stroke and aged over 18 were eligible to participate if the stroke had occurred within the last 42 days and the person had severe upper limb impairment (defined as less than or equal to two on the grasp subsection of the ARAT) [39]. The exclusion criteria were significant musculoskeletal conditions prior to stroke, contra-indications to electrical stimulation, known previous spasticity or hypersensitivity to excipients of BoNTA (onabotulinumtoxin), infection at the proposed injection sites or pregnancy [22].

Participants were screened for the development of spasticity using surface electromyography (EMG) for up to six weeks following stroke. As soon as they were identified to have developed abnormal muscle activity on surface EMG, they were included in the randomised controlled trial.

Randomisation was performed by computer-generated, random permuted blocks in a pseudorandom sequence. A research pharmacist assigned participants according to randomisation and dispensed the appropriate vials of 0.9% sodium chloride solution with or without onabotulinumtoxinA for two clinicians to reconstitute.

Pain was measured at baseline, three months and six months. A 20 cm (0–100) vertical visual analogue scale (VAS) was used to quantify pain on movement of the fingers and wrist; however, due to dysphasia or cognitive impairments, not all participants were able to provide this measure. In order to include the pain response of all participants included in the trial, the results of this VAS data were dichotomised to either having pain or no pain. Participants with a score greater than zero on the scale were dichotomised as having pain. In participants unable to provide a number due to cognition or dysphasia, the presence of pain during the assessment procedure, which involved movement of the arm through range, was identified by either a yes or no response or behavioural cues, e.g., wincing of the face. Consistent with the original study protocol, all pain assessments were carried out by a single blinded independent assessor throughout the study period.

In an attempt to measure the presence of troublesome pain, the numbers of analgesics prescribed were reviewed at each time point. The hypothesis was that the medication would be an indirect reflection of the magnitude of the problem affecting individuals. For analgesic use, the actual number of people in each group and the total number of analgesic medications they were on for each timepoint are reported. The analgesic categories monitored were paracetamol, co-codamol/dydramol, gabanergics, opioids and topical pain medications.

Descriptive statistics are reported for relevant study arm and pain groups. Data that had been dichotomised (people with pain or people with no pain) is presented as the odds ratio (OR) with the 95% confidence interval (CI). Pain prevalence for baseline, three and 6 months is reported as proportions with 95% CI. As this study was not powered for secondary outcome or subgroup analysis, and to mitigate inference errors and multiplicity, significance thresholds were not reported apart from the baseline measures of people with and without pain. An independent Student’s t-test was carried out for comparison of any potential factors that could predict pain between groups of people with and without pain at baseline.

## Figures and Tables

**Table 1:** Frequency of those with pain and without pain at baseline, three and six months.

Time Points of Assessment		Total Sample (*n* = 93)	Group Allocation	
			Treatment (*n* = 45)	Placebo (*n* = 48)
Baseline *	Pain present	No (66)	32	34
		Yes (27)	13	14
Three months	Pain present	No (37)	21	16
		Yes (56)	24	32
Six months	Pain present	No (34)	21	13
		Yes (59)	24	35

* Baseline was a median of 11 days post-stroke (IQR 8 to 19 days).

**Table 2:** Mean difference in people with and without pain at baseline in potential predictors of pain.

Predictor	Pain Baseline	Mean	Std. Dev.	Sig.	Mean Diff.	Lower	Upper
Age (years)	No (66)	68.95	15.081	0.182	4.843	−2.315	12.002
	Yes (27)	64.11	17.392				
Total NIHSS Score	No (66)	15.5	6.127	0.095	−2.352	−5.123	0.419
	Yes (27)	17.85	6.056				
Barthel at Baseline (0–20)	No (66)	3.24	4.568	0.708	0.391	−1.675	2.456
	Yes (27)	2.85	4.512				
Action Research Arm Test Baseline	No (66)	0.82	2.443	0.46	0.374	−0.627	1.374
	Yes (27)	0.44	1.45				

**Table 3:** Mean difference in people with and without pain at 6 months in potential predictors of pain.

Predictor	Pain at 6 Months	Mean	Std. Dev.	Mean Diff.	Lower	Upper
Age	No (34)	67.00	17.934	−0.864	−7.676	5.947
	Yes (59)	67.86	14.661			
Total NIHSS Score	No (34)	14.18	6.474	−3.163	−5.732	−0.59
	Yes (59)	17.34	5.725			
Barthel at Baseline	No (34)	4.18	5.345	1.651	−0.452	3.754
	Yes (59)	2.53	3.91			
Action Research Arm Test Baseline	No (34)	1.47	3.24	1.199	0.038	2.361
	Yes (59)	0.27	1.08			

**Table 4:** Change in reported pain in the sub-group with no pain at baseline.

	Baseline	3 Months		6 Months	
	Number with no pain	Number of new participants reporting pain compared to baseline	OR [95%CI] of not developing pain	Number of new participants reporting pain compared to baseline	OR [95%CI] of not developing pain
Treatment (*n* = 45)	32	11	0.47 [0.17 to 1.26]	11	0.32 [0.12 to 0.89]
Placebo (*n* = 48)	34	18		21	

Odds ratios calculated using number of new participants who develop pain at 3 and 6 months respectively, when compared to baseline participants who had no pain.

**Table 5:** Change in reported pain in the sub-group with pain at baseline for the treatment (BoNTA) and placebo study groups.

	Baseline	3 Months	6 Months
	Number with pain	Number with pain	Number with pain
Treatment (*n* = 45)	13	10	11
Placebo (*n* = 48)	14	13	12

**Table 6:** Numbers of separate analgesic drugs prescribed at discharge, three and six months for the treatment (BoNTA) and placebo study groups.

		Number of Separate Analgesic Drugs Prescribed			
		0 Medications	1 Medication	2 Medications	3 Medications
Discharge	Treatment (*n* = 42)	24	12	6	0
	Placebo (*n* = 46)	18	22	5	1
3 Months	Treatment (*n* = 42)	24	12	6	0
	Placebo (*n* = 44)	13	23	7	1
6 Months	Treatment (*n* = 40)	21	13	5	1
	Placebo (*n* = 43)	12	20	8	3

Denominator for calculating odds ratios is those not on pain medication.

## Data Availability

The original contributions presented in this study are included in the article. Further inquiries can be directed to the corresponding author.

## Abbreviations

The following abbreviations are used in this manuscript:

ARAT	Action Research Arm Test
BoNTA	Botulinum toxin Type-A
CI	Confidence interval
EMG	Electromyography
EUBoSS	Early Use of Botulinum Toxin in Post Stroke Spasticity
IQR	Interquartile range
NHS	National Health Service
NIHSS	National Institutes of Health Stroke Scale
NIHR	National Institute for Health Research
OR	Odds ratio
VAS	Visual analogue scale
